# Big dynorphin is a neuroprotector scaffold against amyloid β-peptide aggregation and cell toxicity

**DOI:** 10.1016/j.csbj.2022.10.014

**Published:** 2022-10-14

**Authors:** Lucía Gallego-Villarejo, Cecilia Wallin, Sylwia Król, Jennifer Enrich-Bengoa, Albert Suades, Marcel Aguilella-Arzo, María José Gomara, Isabel Haro, Sebastian Wärmlander, Francisco J. Muñoz, Astrid Gräslund, Alex Perálvarez-Marín

**Affiliations:** a Unit of Biophysics Dept of Biochemistry and Molecular Biology, Institute of Neurosciences, Universitat Autònoma de Barcelona, Facultat de Medicina, 08193 Cerdanyola del Vallés, Catalonia, Spain; b Department of Biochemistry and Biophysics, Stockholm University, Stockholm, Sweden; c Laboratory of Molecular Biophysics, Department of Physics, University Jaume I, 12071 Castellón, Spain; d Unitat de Síntesis i Aplicacions Biomèdiques de Pèptids, Institut de Química Avançada de Catalunya, IQAC-CSIC, Jordi Girona, 18-26, 08034 Barcelona, Catalonia, Spain; e Laboratory of Molecular Physiology, Faculty of Health and Life Sciences, Universitat Pompeu Fabra, Barcelona, Catalonia, Spain

**Keywords:** Alzheimer’s disease, Amyloid β-peptide, Dynorphins, Peptide therapy, Biophysics

## Abstract

Amyloid β-peptide (Aβ) misfolding into β-sheet structures triggers neurotoxicity inducing Alzheimer’s disease (AD). Molecules able to reduce or to impair Aβ aggregation are highly relevant as possible AD treatments since they should protect against Aβ neurotoxicity. We have studied the effects of the interaction of dynorphins, a family of opioid neuropeptides, with Aβ_40_ the most abundant species of Aβ. Biophysical measurements indicate that Aβ_40_ interacts with Big Dynorphin (BigDyn), lowering the amount of hydrophobic aggregates, and slowing down the aggregation kinetics. As expected, we found that BigDyn protects against Aβ_40_ aggregates when studied in human neuroblastoma cells by cell survival assays. The cross-interaction between BigDyn and Aβ_40_ provides insight into the mechanism of amyloid pathophysiology and may open up new therapy possibilities.

## Graphical abstract

**Figure:**
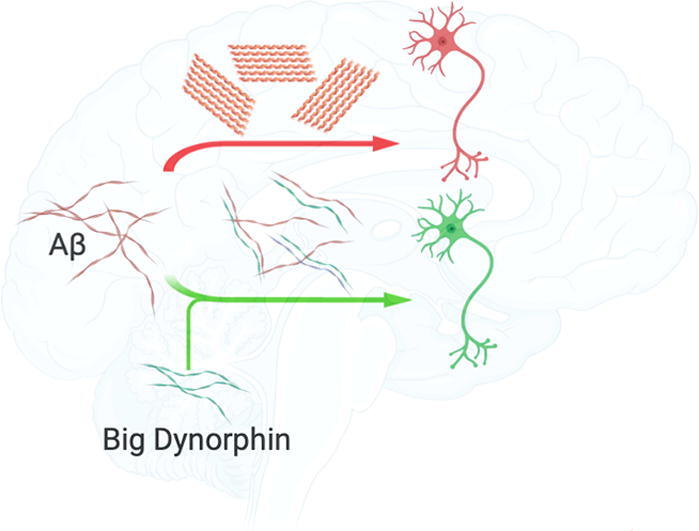

## 1 Introduction

Alzheimer’s disease (AD) is caused by the misfolding of the amyloid-β peptide (Aβ) into β-sheets forming neurotoxic oligomers and fibrils [1], [2]. Therapies directed to inhibit Aβ aggregation and/or to disassembly the aggregated forms are one of the main aims in AD research [3], [4], but most of them are not succeeding [5]. Promising results using an Aβ-binding antibody have been obtained recently in AD patients showing attenuated clinical decline [6].

AD is a multifactorial disease where Aβ plays a key role but other mechanisms also contribute to AD onset and progression, such as the AD risk factor ApoE4 [7]. In fact, the reasons why Aβ starts to aggregate in the brain parenchyma are unknown. The Aβ variants of 40 and 42 residues (Aβ_40_ and Aβ_42_, respectively) have shown different tendency to aggregate, with Aβ_42_ being the most fibrillogenic isoform, but Aβ_40_ is the most abundant in both healthy and AD patients and both types are present in the senile plaques [8]. Metal chemistry has been shown to be of great importance to understand AD [9], [10]. However, it seems that cross-interaction of Aβ with other amyloid and non-amyloid endogenous molecules is opening new ways to develop new diagnostics and therapeutics strategies [11], [12].

Dynorphins are prohormone opioid endogenous peptides derived from prodynorphin (PDYN) [13], which are the canonical substrate for kappa-opioid receptors [14]. Prodynorphin is cleaved at positively charged residues motifs by proprotein convertase (PC2) and other enzymes. It is processed into shorter intermediates, such as big dynorphin (BigDyn, 32 residues), which is further processed into dynorphin A (DynA, 17 residues) and dynorphin B (DynB, 13 residues) [13]. Dynorphins are some of the most positively charged peptides found in our body [15] (Table 1), which makes them highly prone to interact with negatively charged molecules, such as the negatively charged polar head groups of phospholipids [16] and also other molecules, e.g. the Aβ (Table 1).

**Table 1:** Peptide physico-chemical properties.

Peptide	Primary sequence	Mol. weight^a^	pI^a^	Charge^a^	GRAVY Index^a^
Aβ_40_	DAEFR_5_HDSGY10EVHHQ_15_KLVFF_20_AEDVG_25_SNKGA_30_IIGLM_35_VGGVV_40_	4329.8	5.2	−3	0.06
DynA	YGGFL5RRIRP10KLKWD15NQ	2147.5	11.5	+4	−1.26
DynB	YGGFL5RRQFK10VVT	1574.8	11.4	+3	−0.11
BigDyn	YGGFL_5_RRIRP_10_KLKWD_15_NQKRY_20_GGFLR_25_RQFKV_30_VT	3984.7	12.2	+9	−0.98

a Analysis performed using Expasy ProtParam tool[17].

Studies on endogenous opioid systems in AD neuropathology have shown altered μ-, δ-, and κ-opioid receptor binding capabilities [18], [19], [20]. Dynorphins, as substrates for these receptors, have been found to be dysregulated in AD, especially DynA [21]. In addition, AD shows elevated levels of PC2, the enzyme processing PDYN into BigDyn, and further into DynA and DynB [21], [22]. In the initial stages of AD, it has been shown that Aβ oligomers primarily target synapses [23], where altered PDYN processing may lead to changes in dynorphin levels, such as increased DynA presence. Here, we study the cross-interaction between Aβ_40_ and dynorphins based on the premises that: i) DynA is dysregulated in AD [21], ii) dynorphins and Aβ_40_ share the same location at the brain parenchyma, and iii) potential Aβ_40_-dynorphins interactions could be driven by electrostatics and hydrophobicity.

## 2 Materials and methods

### 2.1 Peptide-peptide docking

Dynorphin A, B, and Big dynorphin were modelled in i-Tasser using the DynA 1–13 structure (PDB code 2N2F) [14] and using the secondary structure restraints derived from Hugonin et al. [24]. The structure of choice for Aβ_40_ peptide was PDB code 1BA4 [25]. Peptide-peptide docking was performed in Patchdock [26] and further refined with the Docking2 option in Rosie server [27]. The docking poses were ranked by total score and by interfacial score [28].

### 2.2 Molecular dynamics simulations and analysis

The big dynorphin- Aβ_40_ peptide-peptide complex was prepared and replicated three times in solution for energy minimization and equilibration in CHARMM-GUI [29], using the CHARMM36m force field [30]. The equilibrated output for each complex was reassembled in CHARMM-GUI to produce a complex containing three BigDyn-Aβ_40_ complexes. As control, a system containing three Aβ_40_ peptides was also prepared. Simulations consisted of 5000 steepest descent minimization steps and six NPT equilibration steps in which the restrictions applied on the protein and membrane are released and the timestep gradually increased from 1 fs to 2 fs. MD simulations contained a Parrinello-Rahman pressure coupling and Particle Mesh Ewald for electrostatics and Nose-Hoover for the temperature coupling, extended during 200 ns and at 310.15 K for the production step. Analysis of the trajectories secondary structure conversions was performed using and in-house Python script on the data output from the Timeline plugin in VMD [31].

### 2.3 Peptides

Recombinant Aβ_40_ (with the primary sequence of DAEFR_5_HDSGY10EVHHQ_15_KLVFF_20_AEDVG_25_SNKGA_30_IIGLM_35_VGGVV_40_) and big dynorphin (BigDyn) (primary sequence of YGGFL_5_RRIRP_10_KLKWD_15_NQKRY_20_GGFLR_25_RQFKV_30_VT) were purchased from Alexotech (Umeå, Sweden). Dynorphin A (DynA) and dynorphin B (DynB) were bought from Neosystem Laboratoire (France) with the primary sequences of YGGFL5RRIRP10KLKWD15NQ and YGGFL5RRQFK10VVT, respectively (Table 1). Peptides were also synthesized in-house by solid-phase peptide synthesis (SPPS) as C-terminal carboxamide following a 9-fluorenyl-methoxycarbonyl (Fmoc) strategy. NovaSyn® TGR resin (500 mg, 0.2 meq/g) and Fmoc-protected amino acids (Novabiochem, Merck Millipore, Merck KGaA, Darmstad, Germany) were used. Amino acid side chain protection was effected by the following: triphenylmethyl (Trt) for glutamine and asparagine; *tert*-butyl (tBu) for aspartic acid, and tyrosine; 2,2,5,7,8-pentamethyl-chroman-6-sulfonyl (Pmc) for arginine and *tert*-butoxycarbonyl (Boc) for lysine and tryptophan. The coupling reaction was performed by treatment of Fmoc-amino acids (3 eq.) with 2-(1H-7-azabenzotriazole-1-yl)-1,1,3,3-tetramethyluronium hexafluorophosphate methanaminium (3 eq.) (HATU) (Genscript, Piscataway, NJ, USA) and diisopropylethylamine (6 eq.) (DIPEA) (Fluka-Sigma-Aldrich, St. Louis, MO, USA) in dimethylformamide (DMF) (Scharlau, Barcelona, Spain). The Fmoc deprotection step was performed twice with 20 % piperidine (Fluka-Sigma-Aldrich, St. Louis, MO, USA) in DMF for 10 min. The stepwise addition of each residue was assessed by the ninhydrin test and chloranil test for identification of primary and secondary amines, respectively. The peptides were simultaneously side chain deprotected and cleaved from the resin by treatment with a mixture of trifluroacetic acid (TFA) (Scharlau, Barcelona, Spain), triisopropylsilane (TIS) (Fluka-Sigma-Aldrich, St. Louis, MO, USA) and water (TFA/TIS/H2O, 9.5/2.5/2.5, v/v/v) for 3 h with occasional agitation at room temperature. The solvent was removed in vacuum and the crude peptides were precipitated with diethyl ether (Merck, KGaA, Darmstad, Germany). The solids were dissolved in 30 % acetic acid (Panreac, AppliChem GmbH, Darmstadt, Germany) in water and lyophilized.

The crude peptides were purified by semi-preparative HPLC (1260 Infinity, Agilent Technologies, Santa Clara, CA, USA) in an XBridgeTM Prep BEH130 C18 column (5 μm, 10 × 250 mm, Waters, Milford, MA, USA). The purified peptides were characterized by UHPLC on an Acquity UHPLC (Waters, Milford, MA, USA) chromatograph using an Acquity UHPLC BEH C18 reverse-phase column (2.1 × 100 mm, 1.7 μm particle size). Peptide samples were dissolved in a mixture of acetonitrile (Fisher Scientific, Loughborough, UK) and water (1/1, v/v) and analyzed in the UPLC at a flow rate of 0.3 mL/min. Linear gradients of solvent B (20 mM formic acid in acetonitrile) into solvent A (20 mM formic acid in water) over 10 min at 0.3 mL/min were performed for peptide elution. Both a variable wavelength UV detector and an electrospray ionization mass spectrometry (ESI-MS) were connected to the UHPLC for peptide characterization. UV detection was carried out at a wavelength of 220 nm. ESI-MS was performed with a liquid chromatograph–time of flight (LC-TOF) detector, LCT Premier XE (Micromass Waters, Milford, MA, USA). The mass spectra were recorded in positive ion mode in the *m*/*z* 500–2500 range. The purity of the peptides was higher than 95 % by UHPLC.

### 2.4 Sample preparation

For NMR and ThT kinetics experiments, recombinant non-labeled or uniformly 15 *N*-labeled Aβ_40_ peptides were bought lyophilized from AlexoTech AB (Umeå, Sweden). The lyophilized peptides were stored at −80 °C until used. High-monomer content samples were prepared by dissolving the Aβ_40_ in 10 mM NaOH, pH 12, at a concentration of 1 mg·ml^−1^ and sonicated in an ice-bath for at least three minutes. The peptide concentration was determined by weight or spectrophotometrically by absorbance at 280 nm. Further dilution was done in 10 mM sodium phosphate buffer pH 7.2–7.4. All samples were kept on ice.

All dynorphin peptides were dissolved in Milli-Q water and the concentration was determined by absorbance at 280 nm with an extinction coefficient of 6970 M^−1^·cm^−1^ for DynA and 1280 M^−1^·cm^−1^ for DynB, and 8250 M^−1^·cm^−1^ for Big Dyn.

Aggregated samples for hydrophobicity analysis and cell toxicity measurements were prepared by incubating dynorphins alone or in the presence of Aβ_40_ peptides for 30 h at + 37 °C, representative of a ThT aggregation kinetics end-point (see below). Aggregated mixtures were snap-frozen in liquid nitrogen and kept at −80 °C until further use.

### 2.5 Thioflavin T aggregation kinetics

Prior to kinetic experiments an extra step with size exclusion chromatography (*SEC*) using a Superdex 75 10/300 GL column (GE Healthcare) was performed for the Aβ_40_ sample to remove any pre-formed aggregates [32]. One mg·ml^−1^ Aβ_40_ in 10 mM NaOH or in Gd·HCl (Fig. S3) was injected to the equilibrated *SEC* column and eluted with a flow rate of 0.5 mL·min^−1^ in 10 mM sodium phosphate buffer pH 7.4 at room temperature. The collected fractions were immediately moved to ice. The monomeric peak was collected and the peptide concentration was determined spectrophotometrically by absorbance at 280 nm with an extinction coefficient of 1490 M^−1^·cm^−1^. The Aβ_40_ peptides were further diluted in Eppendorf tubes to 12 μM in 10 mM sodium phosphate buffer pH 7.4 and supplemented with 40 μM Thioflavin T (ThT) as an amyloid probe [33], [34] and different concentrations of DynA, DynB, and BigDyn peptides. The samples were distributed onto a 96-well plate, 100 μL per well, and fluorescence was measured every second minute with a 440 nm excitation filter and a 480 nm emission filter during quiescent conditions at + 37 °C in a FLUOstar Omega microplate reader (BMG LABTECH, Germany). Four replicates per condition were measured. The ThT fluorescence kinetic traces were analyzed using sigmoidal curve fitting according to Eq.1 [35], allowing the parameters aggregation halftime, τ½, and the maximum growth rate, rmax, to be determined.

(1) $$F\left(t\right)=F_{0}+\frac{A}{1+\exp[r_{\max}\left(\tau 1/2\right)=t]}$$

where F0 is the fluorescence signal intensity baseline, A is the fluorescence intensity amplitude, r_max_ is the maximum growth rate and the aggregation halftime, τ½, corresponds to when the monomeric Aβ peptide population is half depleted.

### 2.6 NMR spectroscopy

2D NMR 1H-15 N- heteronuclear single quantum coherence (HSQC) spectra were recorded on a 500- or 700 MHz Bruker Avance spectrometers equipped with cryoprobes at +5 °C. The temperature was chosen due to optimal signal intensity and to avoid Aβ_40_ aggregation. Either DynA, DynB, or BigDyn was titrated onto one sample each with 84 μM monomeric 15N-labeled Aβ_40_ peptides in 10 mM sodium phosphate buffer pH 7.4 (90/10 H2O/D2O). The 15N-labeled Aβ_40_ concentration was determined by weight. The 2D NMR HSQC data was processed with the Topspin version 3.2 software and referenced to the 1H signal of trimethylsilylpropanoic acid (TSP). The Aβ_40_ amide crosspeak assignment in the HSQC spectra was known from previously published work [36].

### 2.7 Reverse phase HPLC

Aggregated peptide mixtures were quenched in 2 % TFA and injected in a Waters 2690 HPLC coupled to a UV detector set to 280 nm. A linear gradient of 25 %-45 % of 0.1 % TFA in acetonitrile was applied for 90 min into a 250x4.6 mm (5 µm) C4 column (Phenomenex) at a flow rate of 0.75 mL/min.

### 2.8 MTT cell viability assays

Aggregated samples of 25 μM Aβ_40_, 10 μM DynA, 10 μM DynB, 10 μM BigDyn and mixtures of Aβ_40_ with each of the Dynorphins were prepared maintaining the concentrations. SH-SY5Y, a human neuroblastoma cell line, was used in the study. Cells were seeded in 96-well plates at density of 10,000 cells/100 μL/well and incubated at 37 °C for 24 h. An Aβ_40_ dose-concentration curve was performed and 3.75 μM Aβ_40_ was chosen for the experiments since they produced a neurotoxicity response around 40 %. Cells were challenged with the different treatments and the toxicity was evaluated after 24 h of incubation. Then 11 µL of 3-(4,5-dimethylthiazol-2-yl)-2,5-diphenyltetrazolium bromide (MTT) solution were added to each well and incubated for 2 h. The reaction was stopped with DMSO and absorbances were measured at 570 nm and 655 nm. Control cells were assumed as 100 % viability. For statistical analysis one-way Analysis of Variance (ANOVA) was performed and corrected by Bonferroni’s multiple comparison test.

## 3 Results

**Fig. 1:**
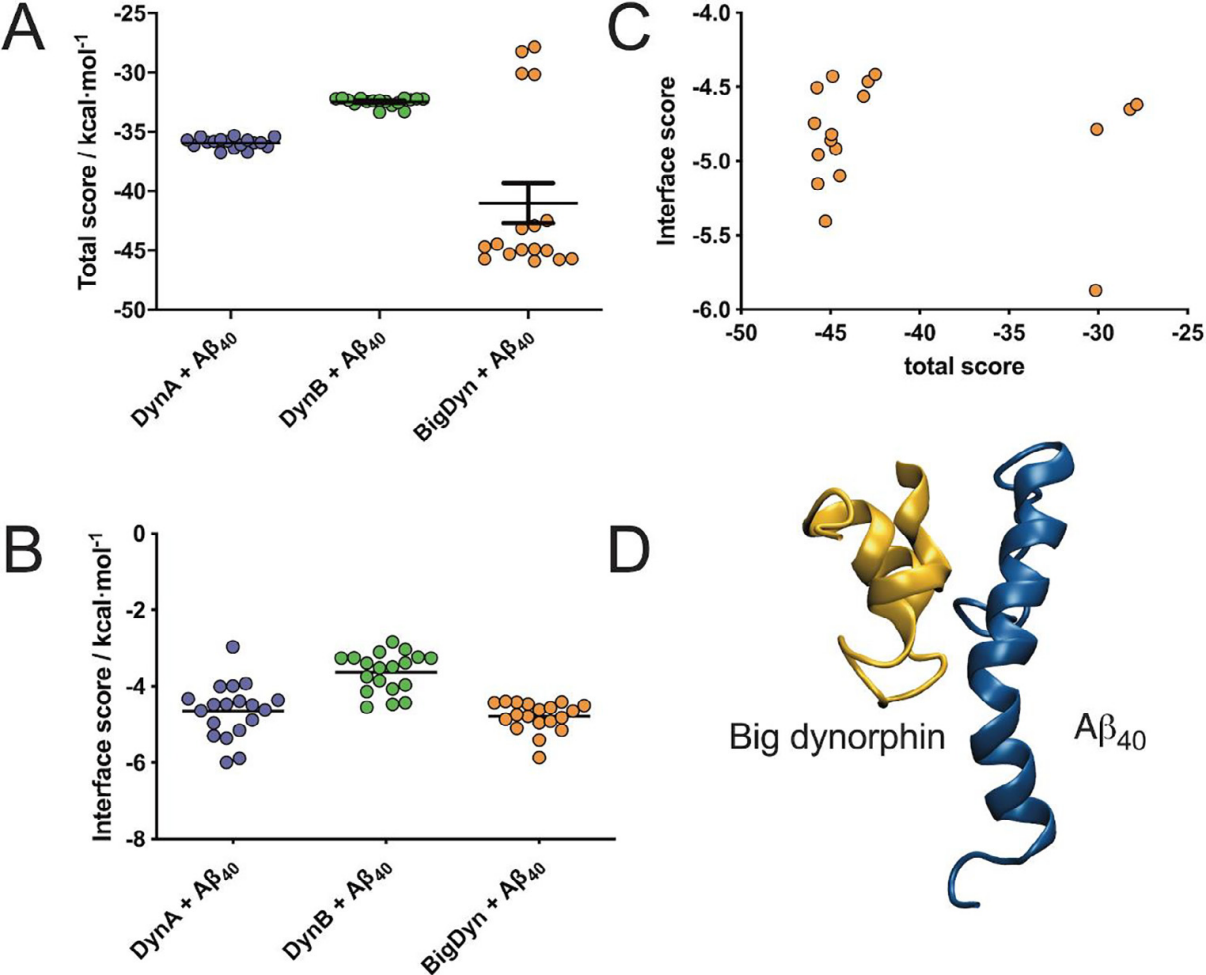
Peptide-peptide docking. A. Global peptide-peptide docking analysis represented by the total score. B. Local peptide-peptide docking analysis represented by the interface score. C. Cross-correlation between total and interfaces scores to select the most-balanced docking solution (indicated by arrowhead). D. Peptide-peptide docking pose of choice based the ratio between total and interface score.

First, to assess the potential interaction between dynorphins and Aβ_40_ we use computational docking (Fig. 1). The peptide cross-interaction is highly likely due to electrostatics, but other physicochemical parameters are relevant, and taken into account in computational docking algortihms such as Rosie [27]. The global docking is assessed by total score (Fig. 1A), but also by the energy of the actual docking interface, namely interface score (Fig. 1B). DynA and DynB show a total score of −35 and –32 kcal/mol, respectively, while BigDyn docking poses are less convergent, but with a lower average total score of −40 kcal/mol. Taking into account both energy terms, the most balanced docking solution corresponds to BigDyn/Aβ_40_ complex, with total score and interface mean score of −40 kcal/mol and −5 kcal/mol, respectively. Thus, to assess the complex stability, the lowest energy BigDyn/Aβ_40_ complex in both terms of global docking and the actual interface docking region (-5.5 interface score; −45 total score in Fig. 1C) is selected as a representative conformation (Fig. 1D).

**Fig. 2:**
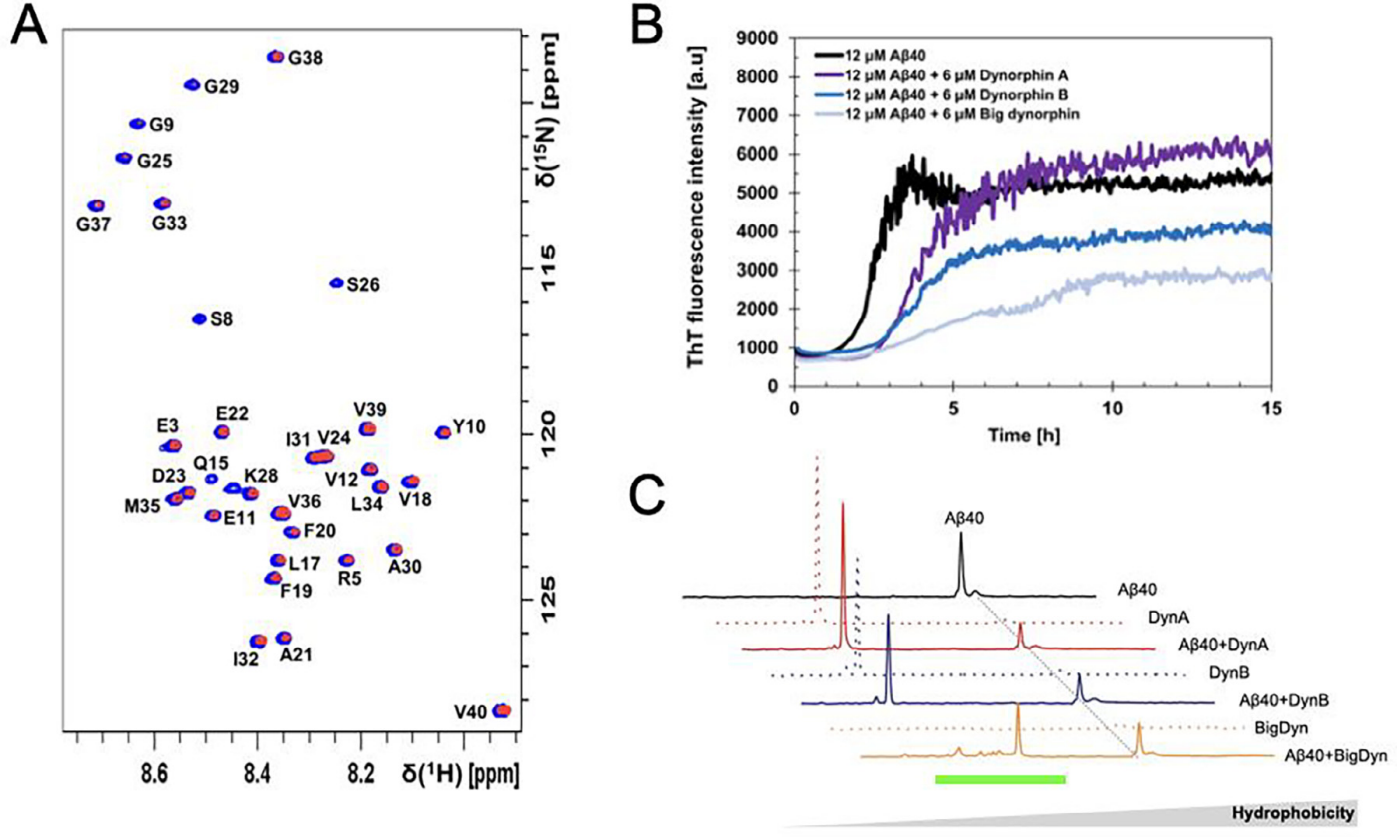
The dynorphin-Aβ_40_ cross-interaction. A. 2D NMR experiments show Aβ_40_ residue-specific perturbations in the presence of big dynorphin (BigDyn). 700 MHz ^1^H-^15^N-HSQC spectra of 84 μM monomeric ^15^N-labeled Aβ_40_ peptide alone (blue amide crosspeaks) and in the presence of 84 μM BigDyn (red amide crosspeaks) in 10 mM sodium phosphate buffer pH 7.4 at + 5 °C. B. Attenuated Aβ_40_ peptide fibrillation kinetics in the presence of dynorphin peptides. 12 µM monomeric Aβ_40_ peptides were incubated in 10 mM sodium phosphate buffer pH 7.4 and 40 µM ThT at + 37 °C under quiescent conditions in the absence and presence of 6 µM dynorphin A (DynA), dynorphin B (DynB) or BigDyn. In the figure the average for each condition calculated from four replicates are shown. The Aβ_40_ peptide stock solution was prepared by size exclusion chromatography (*SEC*) prior to the ThT sample preparation. C. Reverse Phase high pressure liquid chromatography (RP-HPLC) chromatograms for Aβ_40_ + dynorphins mixtures compared to Aβ_40_ alone (25 µM, solid lines) and dynorphins alone (10 µM; dotted lines) incubated during 30 h. The grey dashed line indicates the position of the Aβ_40_ peak, and the green line the position of the peaks specific for Aβ_40_ + BigDyn mixture. Peptide concentrations were kept at 25 µM Aβ_40_, 10 µM dynorphin, and the mixtures at 25:10 µM Aβ_40_:dynorphin.

To validate the dynorphin-Aβ binding interaction we applied 2D NMR experiments to obtain/reveal residue-specific information. Uniformly ^15^N-labeled monomeric Aβ_40_ peptides feature a well-resolved 2D NMR HSQC spectrum. Non-labeled DynA, DynB and BigDyn peptides were sequentially titrated upon a ^15^N-labeled Aβ_40_ peptide sample. Neither the titration of DynA nor DynB showed any specific binding towards the monomeric Aβ peptide, as observed by none-significant signal intensity reduction (data not shown). Titration of BigDyn induced gradual resonance signal attenuations in a concentration-dependent manner (Fig. 2A and Fig. S1). At equimolar BigDyn and Aβ concentrations (Fig. S1) approximately 60 % of the signal intensity had decreased. The loss of signal indicates chemical exchange on the intermediate NMR timescale or potential loss of monomeric peptides into larger structures invisible by solution NMR. Higher concentrations of BigDyn above stoichiometric ratios induced visible precipitation of the sample, arguing for the formation of large BigDyn-Aβ_40_ complexes (Fig. S1). The gradual decrease of signal intensity is uniform and non-specific over the primary peptide sequence, with slightly larger signal attenuation of the N-terminal part of the Aβ peptide.

To obtain further details of the dynorphin-Aβ_40_ interactions, Thioflavin T (ThT) fluorescence labelling was used to study the aggregation kinetics for Aβ_40_ alone and in the presence of dynorphins (Fig. 2B and Table 2). ThT is a small molecule that becomes highly fluorescent when binding to amyloid material like that formed from the Aβ peptides after suitable incubation [33], [34]. It is worth to mention that dynorphins alone did not show any increase of ThT fluorescence intensity after incubation (Fig. S2). DynA incubated with Aβ_40_ increases 0.8-fold the amyloid aggregation level (as shown by the endpoint of ThT fluorescence intensity, Fig. 2B and Table 2). DynB and BigDyn incubated with Aβ_40_ decrease the amyloid aggregation level 1.5-fold and 1.8-fold, respectively. Regarding kinetics, the Aβ_40_ samples incubated with dynorphins show a slower amyloid formation process (as shown by the τ_½_ parameter, Table 2) with 4.4, 3.9, and 5.6 h for the samples incubated with DynA, DynB, and BigDyn, respectively, as compared to 2.5 h for Aβ_40_ alone under the same conditions. The dynorphin-induced slowing down of the Aβ_40_ amyloid formation is also shown in the aggregation kinetics rate (r_max_ in Table 2) with 1.2, 1.7 and 0.5 h^−1^ for DynA, DynB, and BigDyn, respectively, compared to 3.5 h^−1^ for Aβ_40_ alone. Aβ_40_ is prepared as monomer for the ThT experiments (see Material and Methods section for details), we analyzed the anti-aggregation profile of BigDyn against Aβ_40_ nucleated samples, where oligomers and other high molecular species may be present, still showing a slower amyloid formation process (Fig. S3).

**Table 2:** Phenomenological parameters determined from sigmoidal curve fitting of the kinetic traces of amyloid aggregate formation shown in Fig. 2B.

	ThT endpoint fluorescence level^1^	τ_½_ [h]	r_max_ [h^−1^]
Aβ_40_	4600 ± 670	2.5 ± 0.2	3.5 ± 0.9
Aβ_40_ + Dyn A	5500 ± 340	4.4 ± 0.6	1.2 ± 0.3
Aβ_40_ + Dyn B	3000 ± 440	3.9 ± 0.5	1.7 ± 0.8
Aβ_40_ + Big Dyn	2600 ± 310	5.6 ± 1.1	0.5 ± 0.1

1 End point amplitude intensity in ThT kinetics experiments (Fig. 1B).

To assess the role of dynorphins on the nature of the aggregates, hydrophobicity analysis by RP-HPLC (Table 1 for peptides theoretical hydropathy GRAVY indexes) of the peptide mixtures was carried out after a 30-hour incubation (Fig. 2C). Aβ_40_ alone shows a characteristic hydrophobic peak, which is absent in the single dynorphin samples. Aβ_40_ with DynA and DynB mixtures show the chromatogram consisting of the combination of individual peptides, but with lower amyloid content. BigDyn alone is not resolved by hydrophobicity, since it does not show any distinctive peak in the RP-HPLC run, arguing for its high solubility [24]. Aβ_40_ + BigDyn mixture shows a series of peaks corresponding to lower hydrophobicity intermediates compared to the distinctive Aβ_40_ peak (dashed line in Fig. 2C).

**Fig. 3:**
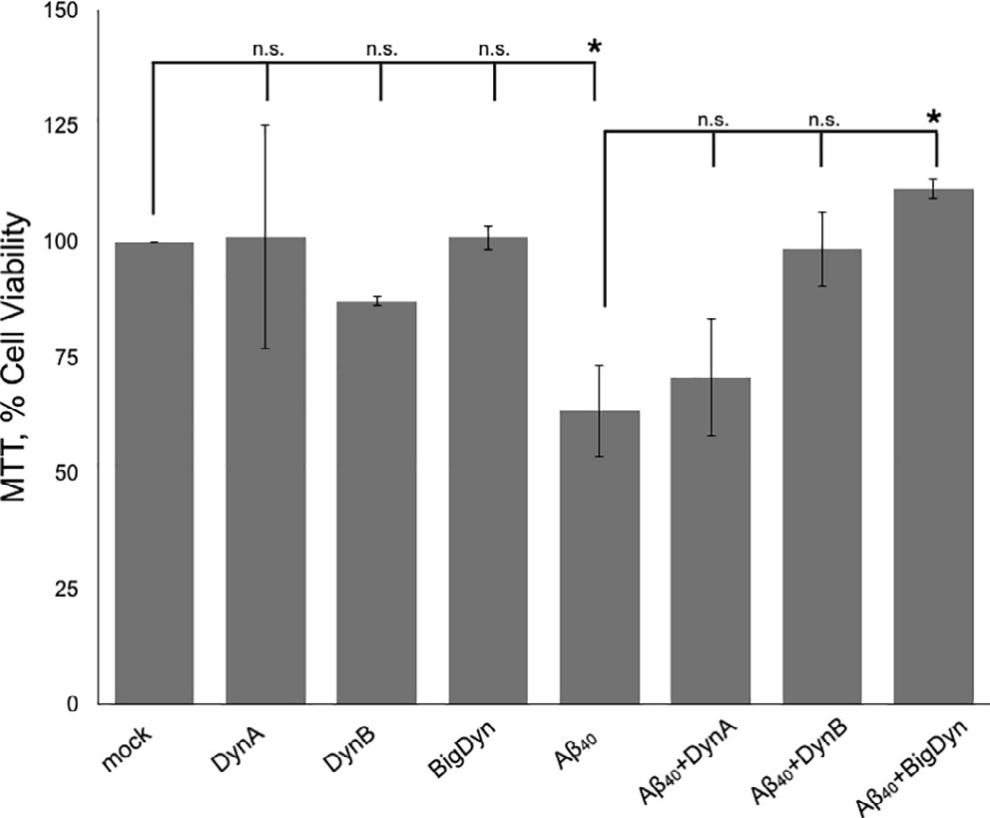
Amyloid-induced cell toxicity in SH-SY5Y cells. The effect on cell viability of Aβ_40_ aggregates and single dynorphins was compared to buffer alone (mock) is represented as the average of at least three independent experiments ± S.E.M. The effect on cell viability of aggregates derived from Aβ_40_ incubated with dynorphins aggregates (Aβ_40_:dynorphin; 3.75:1.5 µM) was compared to Aβ_40_ (3.75 µM). Conditions yielding non-significant and significant (p < 0.05) differences are indicated by n.s. and *, respectively.

The biological effect of the Aβ_40_ + dynorphins mixtures was assessed by the MTT cell viability assay in a human neuroblastoma cell line (SH-S5Y5, Fig. 3). In this setup, the dynorphin peptides alone showed similar viability levels compared to the control (10 mM sodium phosphate buffer pH 7.2). When SH-S5Y5 were treated with Aβ_40_ incubated for 30 h, the viability was reduced to 63.4 ± 9.9 %. Viability levels were: 70.7 ± 12.8 % for Aβ_40_ + DynA and 98.5 ± 8.1 % for Aβ_40_ + DynB. In the case of the Aβ_40_ + BigDyn mixture, the viability was significantly increased to 111.4 ± 2.2 % compared to Aβ_40_ alone (Fig. 3), indicating that BigDyn exerts a cellular neuroprotective effect when incubated with Aβ_40_.

**Fig. 4:**
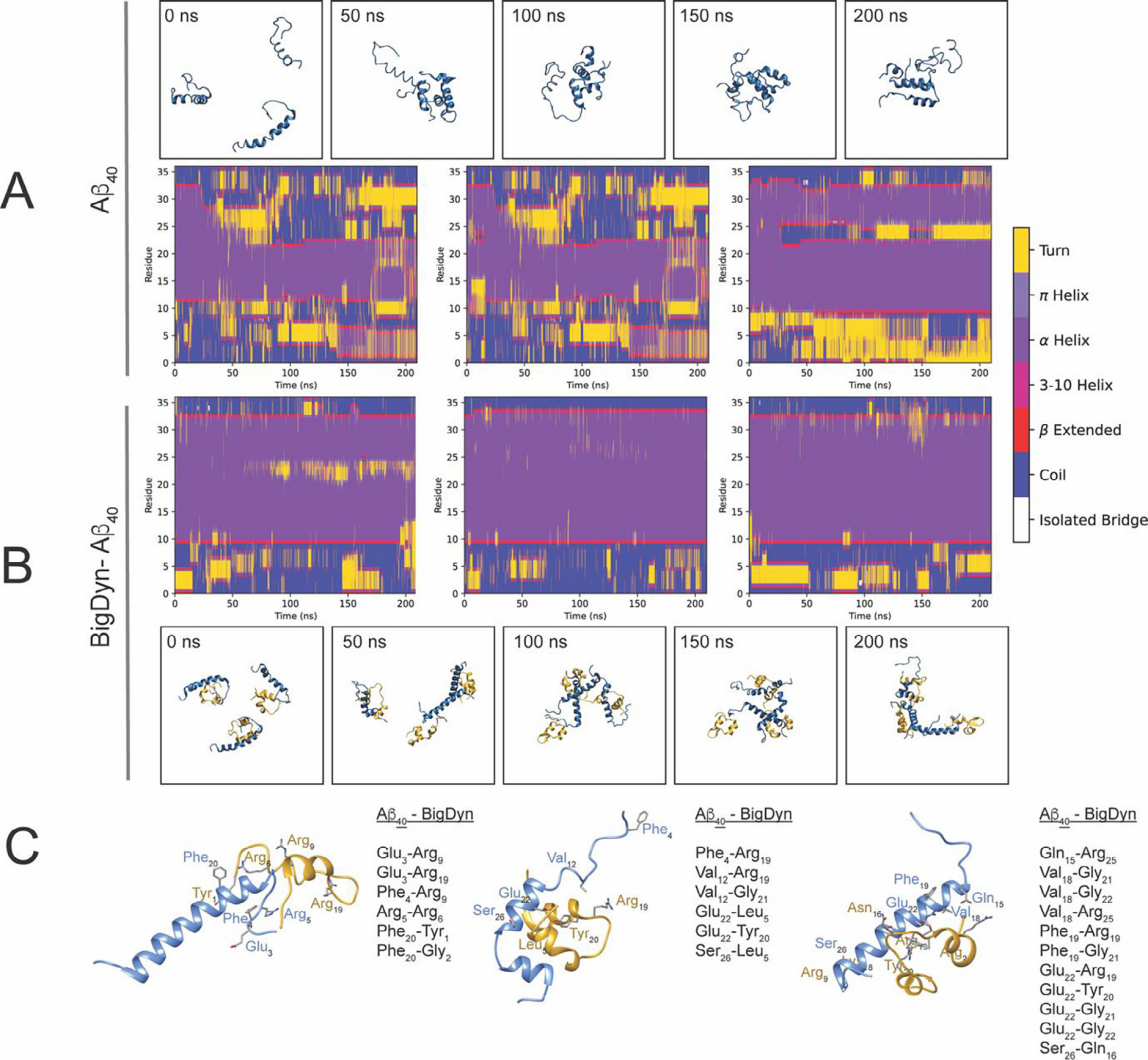
Aβ40 secondary structure conversions induced by the BigDyn cross-interaction. A. Time specific snapshots of the complex of three Aβ_40_ peptides and their respective secondary structure conversions. B. Time specific snapshots of the complex of three Aβ_40_-BigDyn highlighting only the three Aβ_40_ peptides secondary structure conversions. C. Molecular determinants driving the Aβ_40_-BigDyn cross-interaction (blue and gold, respectively), with a total simulation contact time of 90% (left), 89% (middle), and 100% (right). The residues indicated are the ones in close contact (<5Å) for at least 30% of the simulation time (Supporting Table S1).

Although NMR experiments did not show a specific region for the interaction between BigDyn and Aβ_40_ (Fig. S1), we took advantage of the computational setup to gain insight into the molecular determinants of the BigDyn/Aβ_40_ complex. Using the docking pose in Fig. 1D we set the computational system to compare an aggregation-prone situation (three Aβ_40_ peptides randomly placed, Fig. 4A) against three Aβ_40_ peptides complexed with BigDyn (Fig. 4B), as a small representation of the potential interaction landscape of the complex. In the computational setup in Fig. 4A, the three Aβ_40_ peptides quickly interact, and the α-helix structure within the 12–32 residues in Aβ_40_ is rapidly lost towards turn-like and β-extended secondary structures. When Aβ_40_ peptides are complexed with BigDyn the Aβ_40_ cross-interaction process is halted, and the Aβ_40_ secondary structure is stabilized in α-helical secondary structures (Fig. 4B). The peptide-peptide contact is within 89–100 % of the total simulation time (Fig. 4 and Supplementary Table S1) and although the nature of the contacts is diverse, BigDyn residues Arg6, Arg9, Arg19 are prominent in stabilizing not only negatively charged residues such as Glu3 and Glu22 in Aβ_40_ but also aromatic residues, such as Phe4 and Phe20. The BigDyn YGGFL signature present at the *N*-terminus (residues 1 to 5) and in the peptide core (residues 20–24) interact with the 18–26 region in Aβ_40_ (Fig. 4C).

## 4 Discussion

Based on the hypothesis that highly positive peptides such as dynorphins should be able to interact with negatively charged peptides, such as Aβ_40_ we have combined computational and experimental biophysics methods to determine the nature of the interaction and the Aβ_40_ anti-amyloidogenic potential of dynorphins. Then, we have characterized the cytotoxicity of the complexes to evaluate the potential neuroprotective power of dynorphins against Aβ_40_ damage. It has been previously shown that positively charged endogenous molecules such as polyamines and metal ions are able to interfere with Aβ aggregation and toxicity [9], [37], [38].

Here we have used an *in vitro* biophysical and cellular biology methods to confirm and characterize the physical cross-interaction between endogenous opioid peptides, such as dynorphins, and Aβ_40_ peptides. Our results show that dynorphins cross-interact *in silico* with Aβ_40_. Physico-chemical properties such as charge, electrostatic potential, and hydrophobicity favour the interaction as shown by the global, and interfacial scores in docking results, especially for BigDyn. *In vitro*, dynorphins interact with Aβ_40_ early in the aggregation process as shown by ThT aggregation kinetics and monomeric NMR interaction data for BigDyn. DynA and DynB affect the aggregation kinetics without exerting a significant neuroprotective effect in cell viability experiments. BigDyn prevents and slows down the amyloid aggregation of both monomeric and nucleated Aβ_40_ samples. The aggregates derived from the Aβ_40_ and BigDyn cross-interactions are less hydrophobic, and show a neuroprotective behavior in cell viability assays, compared to Aβ_40_ aggregates alone. The neuroprotective mechanism of BigDyn will require further study, because the recovery of cytotoxicity by BigDyn in Fig. 3 could be due to, among other possibilities, the inhibition of monomer to oligomer formation or the binding to oligomers and the hindering their interaction with the cell surface [39], [40], [41], taking into account that BigDyn prevents the aggregation of both monomeric and nucleated Aβ_40_ samples. Among dynorphins, BigDyn appears to be more than the mere combination of DynA and DynB. BigDyn factors such as higher helical content, amphipatic character, size, and positive charge [24] account for a stronger interaction with hydrophobic and negatively charged Aβ_40_. As shown in our MD simulations, Aβ_40_ is prone to aggregate and collapse in water solution. The presence of BigDyn prevents the collapse of Aβ_40_ and the transition to β-strand, stabilizing Aβ_40_ in an α-helical secondary structure through the interaction with hydrophobic residues in the Aβ_40_ disordered *N*-terminus (Phe4, Arg5 and Val12), and the Val18-Phe19-Phe20 hydrophobic core, as shown by short antiamyloid CPP-derived peptides [42]. The 16–22 region of the amyloid peptide has been shown as a key element in the triggering of dimerization process [43]. BigDyn positive residues seem to be key in the interaction with hydrophobic residues, but also with key negatively charged residues in Aβ, such as Glu3 and Glu22, where the latter is a key residue in AD aggregation kinetics and pathology [44].

BigDyn as an intermediate precursor of DynA, appears as an interesting target to decrease pathological DynA levels [21]. Derived from our results, BigDyn may act as an attenuator of cell toxicity and amyloid aggregation, becoming a potential peptidic therapy in AD, by stabilizing Aβ_40_ aggregation in a less toxic or neuroprotective state. Altogether, our results indicate that Aβ_40_ and dynorphins cross-interactions have potential pathophysiological implications in AD, which are worthy to explore further from the therapeutics and pathology perspectives, such as the basis for the design of inhibitory BigDyn-based peptides as therapeutic tools for the treatment of AD. As both the Aβ and dynorphin peptides are known to interact with membranes [45], [46] – Aβ is even produced by enzymatic cleavage of the AβPP (Amyloid-β precursor protein) membrane protein - it appears likely that *in vivo,* the two types of peptides will encounter each other and interact in membrane locations. In the amyloid field, the study of peptide-peptide cross-interactions is key [3], [11], [12], [47], [48] to characterize the physiological environment and determine which players can act as pro-amyloid or anti-amyloid agents opening new therapeutic windows in neurodegenerative disorders, such as studies on the cross-interaction between α-synuclein and endogenous peptides used as peptide-therapeutic scaffolds for Parkinson’s disease [49].

## Funding

This work was supported by the Spanish Ministry of Science and Innovation and Agencia Estatal de Investigación through grant MCIN/AEI/ https://doi.org/10.13039/501100011033 (projects 2019-108434GB-I00 to M.A.-A., PID2020-117691RB-I00 to F.J.M., and PID2020-120222GB-I00 to A.P.-M.), Generalitat Valenciana (project AICO/2020/066 to M.A-A.), FEDER Funds and by the “María de Maeztu Programme” for Units of Excellence in R&D (award CEX2018-000792-M). A.G. was supported by grants from the Swedish Research Council.

## CRediT authorship contribution statement

**Lucía Gallego-Villarejo:** Investigation, Writing – review & editing. **Cecilia Wallin:** Investigation, Writing – review & editing. **Sylwia Król:** Investigation, Writing – review & editing. **Jennifer Enrich-Bengoa:** Investigation, Writing – review & editing. **Albert Suades:** Investigation, Writing – review & editing. **Marcel Aguilella-Arzo:** Investigation, Resources, Writing – review & editing. **María José Gomara:** Resources, Writing – review & editing. **Isabel Haro:** Resources, Writing – review & editing. **Sebastian Wärmlander:** Writing – review & editing. **Francisco J. Muñoz:** Conceptualization, Resources, Writing – review & editing, Funding acquisition. **Astrid Gräslund:** Conceptualization, Resources, Writing – review & editing, Funding acquisition. **Alex Perálvarez-Marín:** Conceptualization, Investigation, Resources, Writing – original draft, Writing – review & editing, Funding acquisition.

## Declaration of Competing Interest

The authors declare that they have no known competing financial interests or personal relationships that could have appeared to influence the work reported in this paper.

## Appendix A Supplementary data

Supplementary data 1 Fig. S1: 2D NMR experiments show concentration dependent Aβ40 peptide perturbations in the presence of BigDyn, Fig. S2: ThT fibrillation kinetics of dynorphin peptides. Fig. S3: Isolation of monomeric Aβ40 and characterization of the ThT aggregation kinetics in the presence of BigDyn. Table S1: Aβ40-BigDyn residue-residue contact list below 5Å cut-off distance derived from the 200 ns molecular dynamics simulations.
