# Supplementary material for: Big dynorphin is a neuroprotector scaffold against amyloid β-peptide aggregation and cell toxicity
Source: Comput Struct Biotechnol J. 2022 Oct 14;20:5672–9. doi: 10.1016/j.csbj.2022.10.014 (PMC9582793; doi:10.1016/j.csbj.2022.10.014)
Supplement: Supplementary data 1 — Fig. S1: 2D NMR experiments show concentration dependent Aβ40 peptide perturbations in the presence of BigDyn, Fig. S2: ThT fibrillation kinetics of dynorphin peptides. Fig. S3: Isolation of monomeric Aβ40 and characterization of the ThT aggregation kinetics in the presence of BigDyn. Table S1: Aβ40-BigDyn residue-residue contact list below 5Å cut-off distance derived from the 200 ns molecular dynamics simulations. [file mmc1.zip › SI_v1.docx]

Supporting Figures


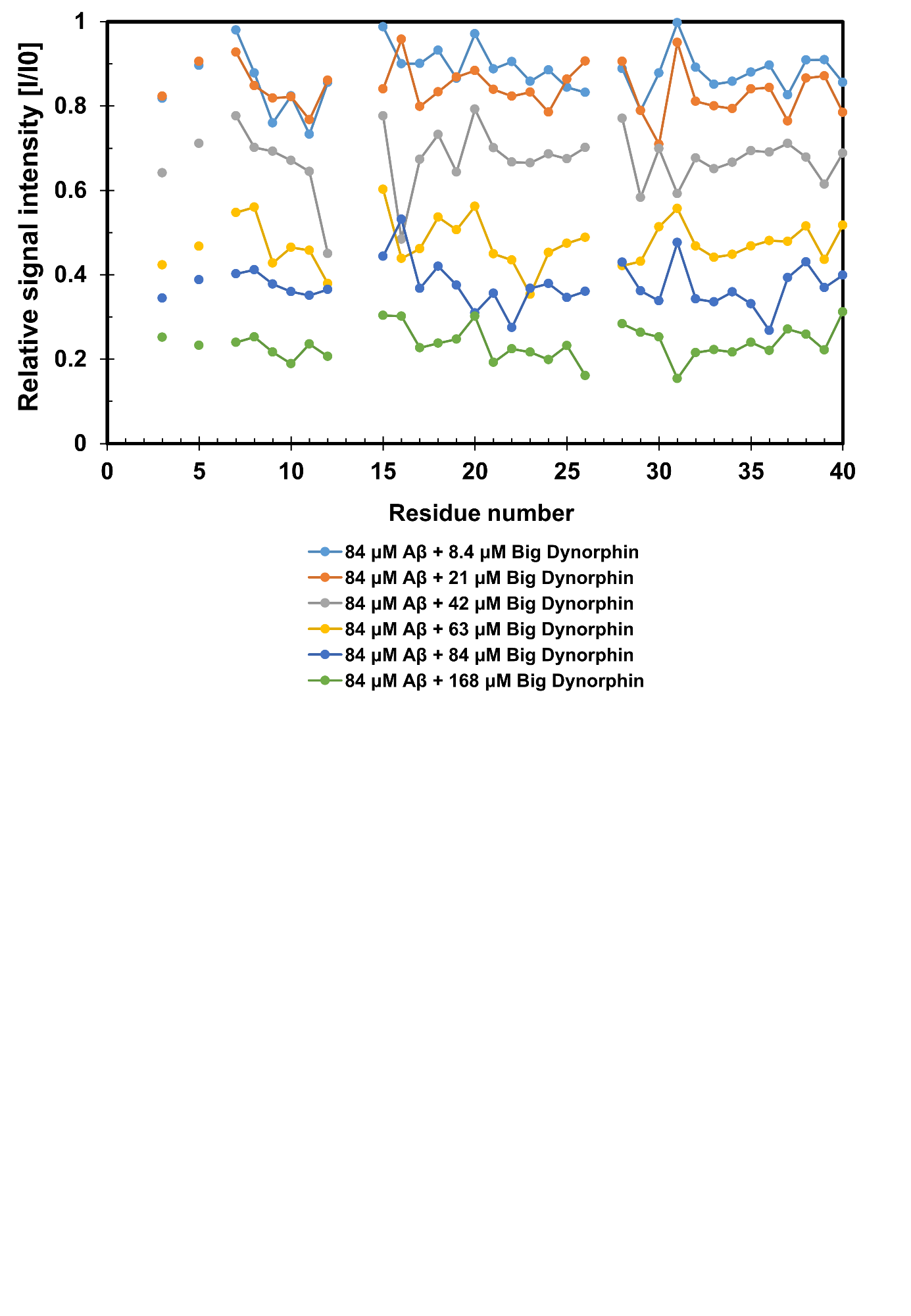


**Figure S1.** 2D NMR experiments show concentration dependent Aβ40 peptide perturbations in the presence of big dynorphin (BigDyn). 700 MHz 1H,15N-HSQC spectra of 84 μM monomeric 15N-labeled Aβ40 peptides in 10 mM sodium phosphate buffer pH 7.4 at +5 °C in the absence and presence of 8.4-168 μM BigDyn were analyzed. The relative signal intensities from the amplitude of the amide crosspeaks were determined and plotted against the primary sequence of the Aβ peptide.


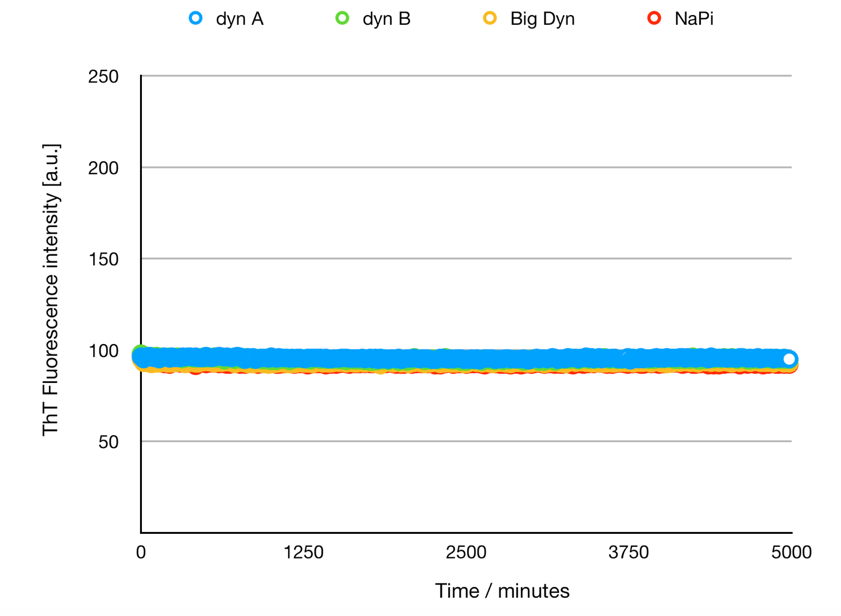


**Figure S2.** ThT fibrillation kinetics of dynorphin peptides. 10 µM dynorphin peptides were incubated in 10 mM sodium phosphate (NaPi) buffer pH 7.4 and 40 µM ThT at +37 °C. In the figure the average for each condition calculated from three replicates are shown.
